# Dopamine D2/3 receptors and functional connectivity during fear extinction: A PET-fMRI study

**DOI:** 10.1016/j.ynirp.2026.100402

**Published:** 2026-09-04

**Authors:** Ashika Anne Roy, Johan Vegelius, Johannes Björkstrand, Mark Lubberink, Mats Fredrikson, Fredrik Åhs, Andreas Frick

**Affiliations:** a Department of Medical Sciences, Experimental Cognitive and Affective Neuroscience Lab, Uppsala University, Uppsala, Sweden; b Department of Psychology, Uppsala University, Uppsala, Sweden; c Department of Psychology, Lund University, Lund, Sweden; d Molecular Imaging and Medical Physics, Department of Surgical Sciences, Uppsala University, Uppsala, Sweden; e Department of Pedagogy, Psychology and Social Work, Mid Sweden University, Östersund, Sweden

**Keywords:** Fear extinction, Dopamine D2/3 receptors, Skin conductance response, fMRI, Brain activation, Functional connectivity

## Abstract

**Background:**

The ability to learn what is dangerous in the environment and what no longer exerts a threat is essential for survival. The latter process is studied using Pavlovian fear extinction, the repeated presentation of a feared stimulus that gradually attenuates the fear response. Dysregulation of the extinction process is implicated in a range of anxiety disorders, yet the neurobiological mechanisms supporting extinction learning in humans remain incompletely mapped, particularly with respect to the modulating role of dopamine.

**Objective:**

To examine the role of baseline dopamine D2/3 receptors in fear extinction and responses to unconditioned stimulus (US) omission.

**Materials and methods:**

Fifteen healthy adults completed a differential cued Pavlovian conditioning paradigm, including one conditioned stimulus (CS+) paired with an aversive electric shock US and one never paired (CS−). Participants underwent [^11^C]raclopride positron emission tomography (PET) of baseline dopamine D2/3 receptor availability prior to fear acquisition, and subsequent fear extinction with skin conductance response (SCR) and functional magnetic resonance imaging (fMRI) continuously recorded.

**Results:**

SCR and neural activations indicated remaining conditioned responding during extinction, with greater responses to CS+ than to CS− in the insula and frontoparietal regions. Higher baseline dopamine D2/3 receptor availability in the amygdala was associated with lower fear-related (CS+> CS−) connectivity between the right amygdala and the right anterior insula. Higher differential SCR (CS+> CS−) was associated with reduced functional connectivity between the right amygdala and left anterior insula. Lower baseline dorsal striatal D2/3 receptor availability was associated with greater mean SCRs to US omission. No associations were detected between baseline D2/3 receptor availability and extinction-related reductions of conditioned SCR or neural responses across trials.

**Conclusion:**

These findings suggest that individual differences in dopamine D2/3 receptor availability are related to threat processing and responses to US omission, but may be less strongly associated with extinction learning itself.

## 1 Introduction

Fear and anxiety are adaptive responses to threat and essential for survival, but they can also cause much suffering when excessive in relation to the threat, as in patients with anxiety disorders (Penninx et al., 2021). According to models of development and maintenance of anxiety disorders, two important processes underlying such excessive fear and anxiety are the learning of fear responses to an innocuous stimulus and deficits in updating responses when the once dangerous stimulus no longer exerts a threat. These processes are investigated experimentally through Pavlovian fear conditioning, by which an initially neutral stimulus (conditioned stimulus or CS+) is repeatedly paired with an unpleasant stimulus (unconditioned stimulus, or US) (Pavlov, 2010). Eventually, the CS+ alone will prompt a conditioned response (CR), which can be measured, for example, by physiological measures such as increased skin conductance responses (SCR) to the CS+ compared to a stimulus never paired with the aversive stimulus (CS−).

Counteracting this fear response is the process of fear extinction, where repeated, non-reinforced exposure to previously conditioned cues progressively attenuates the fear response (Lonsdorf et al., 2017). Fear extinction is a core mechanism in exposure-based therapy approaches, which are widely recognized as the first-line treatment for anxiety disorders (Mcnally, 2007; Milad and Quirk, 2012; Myers and Davis, 2006). However, fear extinction, and likewise exposure therapy, is inherently labile, with fear responses often returning over time (spontaneous recovery), under stress (reinstatement), or with a change of context (renewal) (Bouton, 2004). These findings suggest that extinction does not eliminate the original fear trace, but rather creates a distinct inhibitory memory that constrains fear expression (Bouton, 2002, 2004; Craske et al., 2012, 2018; Maren, 2011). Understanding the process of fear extinction and its neural underpinnings is thus important and may lead to improved treatments of anxiety disorders.

A largely overlapping network of brain regions is involved in fear acquisition and extinction, with the amygdala being critical, as demonstrated in animal studies (J. E. LeDoux, 2000). In humans, the amygdala is less consistently activated, possibly due to methodological issues in both fear conditioning protocols and measurements (Fullana et al., 2018; Radua et al., 2025; Wen et al., 2022). Instead, human neuroimaging studies have implicated an extended network of brain regions in human fear acquisition and extinction, including the amygdala, dorsal anterior cingulate cortex (dACC), anterior insular cortex, thalamus, hippocampus, dorsolateral prefrontal cortex (dlPFC), and midbrain areas like the periaqueductal gray (Agren et al., 2012; Frick et al., 2022; Herry et al., 2007; Radua et al., 2025; Wen et al., 2022) with additional recruitment of the ventromedial prefrontal cortex (vmPFC) (Phelps et al., 2004). Fear extinction thus involves a complex network, demonstrating the intricate coordination required for threat regulation, safety learning, and adaptive emotional responses (Giustino and Maren, 2015; Li et al., 2023; Phelps et al., 2004).

Beyond the neural activation patterns, neuromodulatory systems play a critical role in fear acquisition and extinction. Dopamine and its receptors play a crucial role in the modulation of the amygdala (Heath et al., 2015; Pavlova et al., 2016) and contribute to the formation of fear memories and for encoding new associations (Hamati et al., 2024; Heath et al., 2015; Pavlova et al., 2016; Salinas-Hernández and Duvarci, 2021; Zhang et al., 2025). Till date, most of these research works have been extensively studied in animals; however, there are comparatively limited efforts to translate these findings to humans. Recent studies have shown that dopaminergic pathways influence both the formation and extinction of fear memories in humans. First, endogenous dopamine release during fear acquisition has been linked to facilitation of fear memory formation in the amygdala (Frick et al., 2022), and secondly, exogenous systemic administration of the dopamine precursor L-DOPA has been suggested to enhance extinction memory consolidation (Gerlicher et al., 2019; Kalisch et al., 2019), although findings are mixed (Doubliez et al., 2025). In animals, multiple dopaminergic pathways have been suggested and demonstrated to be involved in and crucial for fear extinction, although the exact nature and contributions of these pathways are yet to be fully elucidated. The pathways include direct modulation of basolateral amygdala (BLA) neurons by ventral tegmental area (VTA) to BLA dopaminergic projections through dopamine D1 receptor signaling (Zhang et al., 2025). Another pathway that facilitates fear extinction involves dopaminergic signaling via D2 receptors in the amygdala, which promotes fear extinction, whereas blocking D2 receptors disrupts fear extinction, as demonstrated in rodents (Shi et al., 2017). Concurrently, dopaminergic modulation within the rodent infralimbic cortex or medial prefrontal cortex (mPFC) facilitates the consolidation of fear-extinction memories, shaping the long-term retention of extinction memory (Sartori et al., 2024).

Moreover, the omission of the aversive outcome during fear extinction can be conceptualized as a reward prediction error, reflecting a discrepancy between the outcome and expectations, and is closely related to dopamine signaling (Schultz et al., 1997). Recent studies have shown that omission of an expected aversive US elicits prediction error related in responses in the VTA/substantia nigra (SN), ventral striatum and frontal cortex, consistent with dopaminergic midbrain involvement during extinction (Thiele et al., 2021; Willems et al., 2025).

In accordance, dopaminergic enhancement of fear extinction in humans involves modulating activity in the ventral striatum/nucleus accumbens, a key region involved in processing reward prediction errors, as well as by strengthening its functional connectivity with the amygdala and vmPFC, which are crucial for fear extinction and retention (Esser et al., 2021). Dopamine also appears to support later stabilization of safety memories; during post-extinction consolidation, enhanced dopaminergic activity promotes the spontaneous reactivation of US omission-evoked vmPFC representations and improves subsequent extinction retrieval (Gerlicher et al., 2019). Thus, dopaminergic signaling is highly important for the extinction and consolidation of fear responses. However, recent pharmacological findings indicate that altering dopaminergic signaling does not uniformly enhance extinction outcomes, suggesting that the contribution of dopamine to human extinction is complex (Doubliez et al., 2025). Extending the brain regions involved, recent studies indicate that during partially reinforced fear acquisition (Ernst et al., 2019) and extinction (Nio et al., 2026), unexpected omission of the US elicits robust activation and connectivity within and between the VTA and bilateral cerebellar Crus I and lobule VI, whereas these responses diminish across extinction trials as US omission becomes expected. This pattern implicates the cerebellum in omission-related prediction-error processing, which may contribute to the updating of threat expectations during extinction, although whether this contribution is dopamine-mediated or through interactions with the VTA remains unclear. Hence, dopamine may contribute to fear extinction both through its role in prediction error signaling that drives extinction learning and through the consolidation of extinction memories.

Despite growing evidence, a central challenge is therefore to identify the neurobiological mechanisms underlying individual differences in fear vulnerability, extinction learning, and, consequently, extinction-learning success. Addressing this requires molecular biomarkers that reflect biologically meaningful variation in brain function. Baseline dopamine D2/3 receptor availability quantified from positron emission tomography (PET) represents one such biomarker and is among the few in vivo methods for quantifying interindividual variation in the dopaminergic system. Preclinical studies have shown that pharmacological blockade of D2 receptors within the infralimbic prefrontal cortex impairs extinction retention and reduces extinction-related neuronal responses (Mueller et al., 2010) and human PET studies indicate that D2/3 receptor availability is linked to individual differences in the functional organization of neural circuits. Striatal D2/3 receptor availability, for example, correlates with variation in frontostriatal activation during response inhibition (Ghahremani et al., 2012). Baseline D2/3 receptor availability is also associated with individual differences in the neural representation of subjective value (Castrellon et al., 2019), as well as with motivational anhedonia and antidepressant treatment outcomes in major depressive disorder (Peciña et al., 2017). Furthermore, D2 receptor availability is related to interindividual variability in reward-related neural processing, with midbrain D2 receptor availability predicting the strength of ventral striatal representations of expected reward value (Dang et al., 2018). Hence, translating these findings to humans and investigating the contribution of baseline dopamine D2/3 receptor availability to fear extinction in humans is essential to fully understand the neural underpinnings of adaptive learning of emotional responses. In addition, given that reward-like prediction errors from unexpected US omission drive extinction learning, individual differences in D2/3 receptor availability may cause variability in US omission-related dopaminergic signaling, prediction-error processing, and extinction success in humans.

The present study examined the role of dopamine D2/3 receptors in fear extinction in healthy subjects by combining [^11^C]raclopride PET of dopamine D2/3 receptors prior to fear acquisition with functional magnetic resonance imaging (fMRI) and SCR during fear extinction. We targeted both conditioned responses (CS+ − CS−) and responses to unexpected omissions of the US.

For extinction-related neural responses, we hypothesized that individual differences in baseline dopamine D2/3 receptor availability would explain variability in conditioned responses and their reductions across extinction trials, although we had no hypothesis regarding the direction. Additionally, we tested whether SCR of conditioned responses were associated with corresponding functional brain activations and connectivity. We also explored the temporal dynamics of these associations, which currently remain poorly understood.

For the US omission responses, we tested the SCR and neural responses, its temporal change over extinction trials, and its association to baseline D2/3 receptor availability.

## 2 Materials and methods

### 2.1 Participants and procedure

Fifteen individuals (mean ± SD age 25 ± 5.2 years; 9 women, 6 men; 14 right-handed, 1 left-handed) recruited through local advertisements participated in the study. All individuals included in the study were self-reported healthy, non-smokers who did not use any drugs, and written consent were provided by them. The sample consisted of the subset of participants from (Frick et al., 2022) who, in addition to fear acquisition, underwent fear extinction during fMRI on the same day. The study was conducted in accordance with the Helsinki Declaration and approved by the Regional Ethics Committee in Uppsala and the Radiation Safety Committee at Uppsala University Hospital (Dnr, 2016/102, date April 6 2016 and amendment on May 24, 2017). All participants provided written informed consent prior to participation. The participants underwent a standardized procedure including 90-min continuous PET scan using bolus + infusion intravenous delivery of [^11^C]raclopride, fear acquisition and extinction. Following the initial 50-min baseline period of PET acquisition, participants underwent a differential cued fear acquisition paradigm lasting 20 min, followed by a 20-min rest, for a total of 90 min of PET acquisition. After the PET scan, participants underwent fear extinction, with simultaneous BOLD fMRI and psychophysiological responses via skin conductance recording. Upon completion of extinction learning, participants exited the scanner and their contingency awareness was assessed using a response to a 0-100 scale, to rate how certain they were of receiving a shock (US) when viewing the CS+ and the CS− during the experiment. Evidence for contingency awareness was evaluated using a paired-sample *t*-test comparing CS+ and CS− discrimination ratings. As the fear acquisition data have already been published (Frick et al., 2022), we here focus on the baseline PET measure of dopamine D2 receptors and its association with fear extinction and US omission.

### 2.2 Fear acquisition and extinction paradigm

The fear acquisition paradigm was assessed during a 20-min session comprising 40 trials, with 20 presentations of each neutral geometric cue (brown arrow; blue circle). One stimulus was designated as the CS+ and co-terminated with a 200-ms electric shock (US) on 80% of trials (partial reinforcement; 16 out of 20 pairings), while the CS− was not coupled to any shock. US intensity was individually calibrated using a staircase procedure to reach a level perceived as aversive but tolerable. To limit potential confounding influences, conditioned stimuli were counterbalanced across participants. The fear extinction task also lasted 20 min and comprised 20 presentations each of CS+ and CS− without the US. Each CS was displayed for 6 s, with an inter–trial interval (mean = 24.3 s; range 21.8-27 s) marked by a fixation cross. The tasks were implemented using E−prime 2 (Psychology Software Tools, Pittsburgh, PA, USA) and displayed on a 32-inch computer screen, viewed through a mirror attached to the head coil. A SyncBox (NordicNeuroLab, Bergen, Norway) synchronized the temporal alignment between task onsets with fMRI acquisition.

Electric shocks (US) were administered to the right dorsal lower arm of the subject using disposable radio-translucent electrodes (EL509, BIOPAC Systems, Goleta, CA, USA). The US was generated through an STM100C interface linked to the STM200 constant-voltage unit and controlled by the BIOPAC MP150 system (Biopac Systems, Goleta, CA, USA). Full details on the fear acquisition task and the results from this have been published previously (Frick et al., 2022).

### 2.3 Skin conductance responses

Skin conductance activity was acquired throughout the fear acquisition and extinction tasks using a BIOPAC MP150 (BIOPAC Systems, Goleta, CA, USA). Signals were obtained with a radiotranslucent disposable Ag/AgCl electrodes (EL509 Biopac electrodes) preloaded with isotonic electrode gel (GEL101 Biopac gel), positioned over the left hypothenar eminence. Slow baseline drift and high frequency noise were removed from the SCR signal using 0.05 Hz high-pass and 10 Hz hardware low-pass filters, respectively.

The skin conductance data were analysed using a standardized custom-written in-house MATLAB (Version 9.13.0.2080170, R2022b; The MathWorks, Inc., Natick, Massachusetts, USA: https://www.mathworks.com) script implementing the preprocessing procedures described below, consistent with established physiological methods (Boucsein et al., 2012) and prior human fear-conditioning research studies (Milad et al., 2007). Initially, a 10 ms median filter was applied, followed by low-pass filtering using a first-order Butterworth filter with a cutoff frequency of 5Hz. The data were subsequently down-sampled to 10 Hz. SCR were defined for each CS trial during acquisition and extinction, as well as for the omission of US during extinction. For SCR analyses, we applied an amplitude threshold of 0.01 μS and trials not meeting these criteria were scored as 0, whereas amplitudes exceeding 5 μS were coded as missing. A square-root transformation was applied to the preprocessed, baseline corrected SCR amplitudes to minimize data skewness and inter-individual variability.

For each CS trial, the maximum peak skin conductance level occurring at 1 to 6s after CS onset was calculated, from which the mean skin conductance level computed during the 1s interval preceding CS stimulus onset from the peak amplitude. To quantify SCRs associated with US omission during extinction, the mean skin conductance level of the last 1s of each CS presentation was subtracted from the maximum skin conductance level observed within 4s after each CS offset.

SCR data were analysed trial-by-trial and by aggregating the data into indices for each phase. The differential SCR between CS+ and CS−), which is an index of fear expression was calculated for both acquisition and extinction separately can be expressed as:

(1) $$\Delta {\text{SCR}}_{All\;Trials}={\text{SCR}}_{CS+}-{\text{SCR}}_{CS-}$$

Where,

SCR_CS+_ is the mean skin conductance response during the presentation of the 20 CS+ stimuli, SCR_CS-_ is the mean skin conductance response during the presentation of the 20 CS− stimuli.

We also computed the differential response over time between the CS+ and CS− for early (first 5 trials) and late (last 5 trials) in the extinction phase, with the difference between these early and late differentials being used to evaluate temporal changes in physiological responses during fear extinction. This can be mathematically expressed as,

(2) $$\Delta {\text{SCR}}_{Early-Late}=Early\left({\text{SCR}}_{CS+}-{\text{SCR}}_{CS-}\right)-Late\left({\text{SCR}}_{CS+}-{\text{SCR}}_{CS-}\right)$$

Where,

$Early\left({\text{SCR}}_{CS+}-{\text{SCR}}_{CS-}\right)$ and $Late\left({\text{SCR}}_{CS+}-{\text{SCR}}_{CS-}\right)$ are the mean SCR values during early (first 5) and late (last 5) trials, respectively.

For US omission, we calculated the mean differential SCR (noUS CS+ − noUS CS−) for both the first 5 trials, where expectation violation should be the greatest, and for all trials, both used subsequently as indices of prediction error.

#### 2.3.1 Skin conductance response statistics

First, to determine fear acquisition, we fitted a linear mixed-effects (LME) model with the lme4 package (Bates et al., 2015) in R to the SCR during the fear acquisition phase. The model included stimulus type (CS+ or CS−), trial number (1–20), and their interaction as fixed effects, with participant as a random intercept. We also tested for overall fear expression during acquisition using the differential SCR (CS+ − CS−) for all trials. Furthermore, we investigated whether interindividual variability in baseline dopamine D2/3 receptor availability in the amygdala, dorsal striatum, and ventral striatum was associated with the magnitude of conditioned fear acquisition.

We then focused on the extinction phase, and fitted trial-level SCRs in a LME model. Similar to the fear acquisition model, the extinction model included stimulus type (CS + or CS−), trial number (1–20), and their interaction as fixed effects, with participant as a random intercept. We then entered baseline dopamine D2/3 receptor availability from the amygdala, dorsal striatum, and ventral striatum to the model to examine relationships between these and extinction.

We also used the aggregated measure of SCR to test whether overall differential SCR (CS+ − CS−) during extinction, a measure of fear expression, significantly differed from zero using a two-tailed one-sample *t*-test, separately for fear acquisition and extinction learning. Cohen's *d*_*z*_ was calculated to estimate effect size, and 95% confidence intervals (CI) were reported.

To determine whether baseline D2/3 receptor availability in the amygdala, dorsal striatum, and ventral striatum was associated with initial fear expression, residual fear, or changes across extinction, correlations were computed between baseline D2/3 availability and differential SCR during early extinction (trials 1-5), late extinction (trials 16-20), and extinction magnitude ($\Delta {\text{SCR}}_{Early-Late}$) respectively.

For SCRs to unexpected US omission, we fitted trial-level SCRs in a LME model with stimulus type (US CS+: or noUS CS−), trial number (1–20), and their interaction as fixed effects, and participant as a random intercept. In addition, we tested whether D2/3 receptor availability was associated with aggregated measures of US omission-related SCR (noUS CS+ − noUS CS−), both for the first 5 trials and for all trials.

Bivariate associations were tested using Pearson correlation coefficients (r), and corresponding two-tailed p-values were calculated. For significant associations, robust correlation was assessed using the percentage-bend correlation method (pbcor function; WRS2 package v1.1.7 Mair and Wilcox, 2019). The R version 4.4.2 (R: The R Project for Statistical Computing, n.d.) was used for all these analyses.

### 2.4 PET and fMRI image acquisition

To examine dopamine D2/3 receptor availability, subjects underwent a 90 min dynamic PET scan on a 3 T Signa PET-MR scanner (GE Healthcare, Waukesha) with an 8-channel head coil. The PET scan was initiated simultaneously with the start of a [^11^C]-raclopride bolus + infusion protocol (total amount of radioactivity mean ± SD 516.6 ± 91.4 MBq; kbol 107 min) and, here we used the initial 50 min of the PET data collected before the fear acquisition. Foam padding was used to minimize the subject's head movement during scanning.

During the 50 min baseline before fear acquisition (approximately 15 min after bolus injection), high-resolution 3D T1-weighted structural images were collected with an eight-channel coil, yielding 146 slices. The repetition time (TR), echo time (TE), inversion time, flip angle, and slice gap were 8.6 ms, 3.3 ms, 450 ms, 12°, and 1.2 mm, respectively. Images were acquired with 1.2 ×1.2 × 1.2 mm^3^ voxel resolution on a 256 × 256 acquisition matrix.

Single shot echo-planar imaging (EPI) with the following parameters (TR = 3000 ms, TE = 30ms, flip angle = 90°, Acquisition matrix = 64 × 64, voxel size = 3.0 × 3.0 × 3.0 mm^3^, slice gap = 3.4 mm, 45 axial slices), acquired in interleaved bottom-up order, was used to collect blood-oxygenation-level dependent (BOLD) fMRI for investigation of brain activation and connectivity during the fear extinction task.

### 2.5 PET analysis

#### 2.5.1 PET modeling and quantification

Dynamic PET images were reconstructed as 18 consecutive 5-min dynamic frames using an ordered-subsets expectation-maximization (OSEM) algorithm (4 iterations with 28 subset partitions), incorporating resolution recovery and 5 mm Gaussian post-filter.

Quantitative PET images were reconstructed using an atlas-based method for attenuation correction, followed by other standard corrections necessary for analysis. Frame-to-frame motion was corrected using rigid body realignment correction using the Voiager software (GE Healthcare, Uppsala, Sweden). T1-weighted MRI images from all participants were co-registered to their corresponding summed PET image. Subsequently, the images were segmented into gray matter, white matter, and cerebrospinal fluid (CSF) using SPM8 (SPM - Statistical Parametric Mapping, n.d.). Non-displaceable binding potential (BP_ND_) was quantified at the voxel level by modeling the first 50 min of time-activity curves (TAC). A basis-function implementation of the simplified reference tissue model (SRTM) framework was applied, using cerebellar gray matter as the reference region. The analysis resulted in parametric images of [^11^C]raclopride BP_ND_, where each voxel represents the quantitative measure of dopamine receptor D2/3 availability.

#### 2.5.2 PET parametric image preprocessing

Parametric PET images were normalized to the Montreal Neurological Institute (MNI) reference space using SPM12 by an indirect approach, involving initial co-registration to the corresponding T1-weighted MRI followed by normalization to the MNI template. The resulting deformation fields were subsequently applied to the co-registered PET parametric image, which was resampled to an isotropic voxel resolution of 2 mm. Finally, spatial smoothing was performed by convolving the data with an isotropic Gaussian kernel with a full-width-at-half-maximum (FWHM) of 8 mm. Masks for three regions of interest (ROIs) (amygdala, dorsal striatum and ventral striatum) were defined from the Harvard-Oxford subcortical structural atlas and Oxford-GSK-Imanova structural striatal atlases (Tziortzi et al., 2011), both implemented in FMRIB Software Library (FSL v6.0.6.4) (Jenkinson et al., 2012). Mean BP_ND_ within each ROI was extracted and used in subsequent analyses.

### 2.6 fMRI analysis

The raw DICOM images were pseudonymized, converted to NIFTI format, and stored in accordance with the Brain Imaging Data Structure (BIDS) format (Boré et al., n.d.). Analyses of fMRI data were performed using CONN(Whitfield-Gabrieli and Nieto-Castanon, 2012) (RRID:SCR_009550) release 22. b and SPM12 (Penny et al., 2007) (RRID:SCR_007037) release 12.7771 within MATLAB. Prior to analysis, structural T1-weighted MRI were visually inspected for quality, followed by assessment of fMRI head motion using fsl_motion_outliers (Jenkinson et al., 2012) with a 0.5 mm framewise displacement (FD) (Power et al., 2011); on average 2.61 ± 2.49% of volumes were identified as outliers across participants (range: 0 – 8.35%).

Additionally, task event logs generated in E-Prime were also converted into BIDS-formatted tsv files using custom bash scripts and adapted accordingly to later build first-level general linear modeling(GLM) regressors in SPM and CONN toolboxe.

#### 2.6.1 fMRI activation analysis

##### 2.6.1.1 Preprocessing

Preprocessing and fMRI data analysis were carried out using the SPM12 toolbox. The T1-weighted images were checked for MR artefacts, motion, and structural abnormalities using a systematic quality check. The T1w anatomical image was reoriented to align with the anterior commissure – posterior commissure (AC–PC) using acpcdetect v2 (Ardekani and Bachman, 2009) before segmentation and normalization. Each subject's fMRI volumes were realigned and corrected for slice timing difference, coregistered with the T1w anatomical data, spatially normalized to the MNI template (using the deformation field calculated during the segmentation step of the T1 anatomical volume), and smoothed using a 6 mm^3^ Gaussian Kernel.

##### 2.6.1.2 GLM model fitting

For each participant, event-related responses to the CS (CS+ and CS−) were modeled in SPM12 within a GLM framework by convolving the event onsets and durations (6 s) with the canonical hemodynamic response function (HRF), while including the six motion realignment parameters and spike regressors as nuisance regressors.

In addition, to model US omission-related BOLD responses, neural responses associated with unexpected US omissions following both CS+ and CS− were time-locked to the CS offset. These omissions were modeled as separate delta functions (zero-duration) within the GLM.

The BOLD (CS+ > CS−) aggregated contrast tested differential responses to the conditioned cue at CS onset. To test whether differentiation was greater at the beginning of extinction learning, CS+ and CS− trials were additionally modeled for the first and last five trials, using the contrast early (CS+ > CS−) > late (CS+ > CS−).

To characterize trial-wise extinction learning trajectories, the neural dynamics underlying the progression of fear extinction learning were examined using a parametric modulation (PM) approach implemented in SPM12. For each participant, CS+ and CS− event onsets were modeled as separate regressors in the first-level GLM, each accompanied by a first-order parametric modulator indexing mean-centered trial number (1–20) to capture linear changes in BOLD responses across the extinction learning phase.

Expectancy violation during extinction was examined using the aggregated omission contrast (noUS CS+ > noUS CS−) for all trials. To isolate the trials associated with greatest expectancy violation, the same contrast was additionally restricted to the early (1–5 trials) US omission trials. In addition, we tested if responses to the unexpected US omission changed over the course of the extinction phase by using trial number as a parametric modulator on the omission regressors.

##### 2.6.1.3 Statistics

We conducted whole-brain voxel-wise group-level analyses on the contrasts of interest from the first level models described above. First, the aggregated differential BOLD (CS+ > CS−) contrast was used to examine group level fear responses. In a separate model, we examined trial-by-trial (parametric modulation) extinction trajectories (CS + PM > CS− PM). Finally, we tested the US omission-related contrast (noUS CS+ > noUS CS−). We then tested whether these contrasts were associated with differential SCR (CS+ − CS−) and baseline amygdala, ventral striatum, and dorsal striatum D2/3 availability. Second-level analyses were conducted in SPM12 using a one-sample *t*-test, including SCR and baseline D2/3 in separate models, and applying a voxel-level uncorrected threshold of p < 0.001 to define clusters, followed by cluster-level inference at p < 0.05.

#### 2.6.2 fMRI connectivity analysis

##### 2.6.2.1 Preprocessing

Functional and anatomical data were preprocessed using the CONN toolbox (default_MNI pipeline). Volumes exceeding 0.5 mm FD were classified as outliers using the Artifact detection tools (ART) (Power et al., 2011). The functional data were smoothed using spatial convolution with a Gaussian kernel of 6 mm FWHM. The data was then denoised using anatomical component-based noise correction (aCompCor), followed by high-pass frequency filtering of the BOLD timeseries above 0.01 Hz. From the number of noise terms included in this denoising strategy, the effective degrees of freedom of the BOLD signal after denoising were estimated to range from 289.5 to 358.1 (average 329.6) across all subjects.

##### 2.6.2.2 Generalized psychophysiological analysis

The generalized psychophysiological interaction (gPPI) model was used to examine task condition-specific changes in functional connectivity between brain regions (seed and targets) identified in previous studies as involved in fear extinction. Spherical masks of the seed ROIs were created for the vmPFC, dACC, bilateral amygdala, and anterior and posterior insula, using MNI coordinates from previous studies. The dACC and vmPFC regions were defined with an 8 mm radius sphere centered at MNI coordinates (2,22,29) and (5,35, −13), respectively, based on prior studies of fear and emotion regulation (Linnman et al., 2012). The bilateral amygdala with a 3 mm radius (left: −23, −5, −18, right: 23, −4, −18) was defined using the CONN atlas and corresponds to the regions derived from the Harvard-Oxford structural atlas. Similarly, spherical masks of insular subregions with a radius corresponding to 5 mm for both anterior (left: −33,13, −7; right: 32,10, −6) and posterior (left: −38, −6,5; right: 35, −11,6) were also defined (Deen et al., 2010).

For the US omission connectivity analyses, we focused on the VTA and cerebellum identified in a recent study (Nio et al., 2026). ROIs were defined using the AAL3 atlas (Rolls et al., 2020) to define separate bilateral cerebellar (lobule VI, Crus I, and Crus II) and VTA masks.

The gPPI model included physiological factors (BOLD signals from the *a priori* defined ROI regions), psychological factors (boxcar regressors modeling conditions following convolution with canonical HRF) and their interaction terms reflecting psychophysiological interaction (PPI) terms. ROI to ROI functional connectivity changes between conditions were characterized by the multivariate regression coefficient of the psychophysiological interaction terms in each model.

The same contrasts as in the activation analyses were tested. We first used the aggregated mean measure to test the functional connectivity across the main effect of stimulus type (CS+ − CS−). To capture the extinction learning trajectory, we tested the parametric modulation of connectivity (CS + PM > CS− PM) over extinction trials, reflecting trial-wise decline in differential response.

Finally, we examined whether cerebellar-VTA functional connectivity was related to unexpected US omissions, first across all trials and then assessing the change in connectivity by entering trial number as a parametric modulator of the US omission events (noUS CS+ > noUS CS− × Trial number).

To investigate whether the SCR and baseline D2/3 receptor availability of the amygdala, dorsal and ventral striatum were related to ROI-ROI (vmPFC, amygdala, dACC, and anterior and posterior insula) fMRI connectivity during fear extinction learning, each predictor was tested separately against the differential connectivity (CS+ > CS−) and the extinction trajectory (PM approach).

For the US omission contrast, baseline D2 receptor availability was used as a predictor of cerebellum-VTA connectivity for both all trials and in the trial-wise parametric modulation (noUS CS+ > noUS CS−).

##### 2.6.2.3 Statistics

Inferences were performed at the connection-level uncorrected threshold of p < 0.001, and statistical significance was evaluated using FDR (Benjamini and Hochberg, 1995) correction at p < 0.05, applied across all ROI-to-ROI connections. Parallelly, cluster-level inference on groups of functionally and anatomically similar connections identified using hierarchical clustering across the ROI-to-ROI connectivity matrix was conducted. All ROI definitions were drawn from *a priori* defined regions, and connectivity maps are reported in MNI space.

## 3 Results

### 3.1 Skin conductance responses

#### 3.1.1 Fear acquisition

**Fig. 1:**
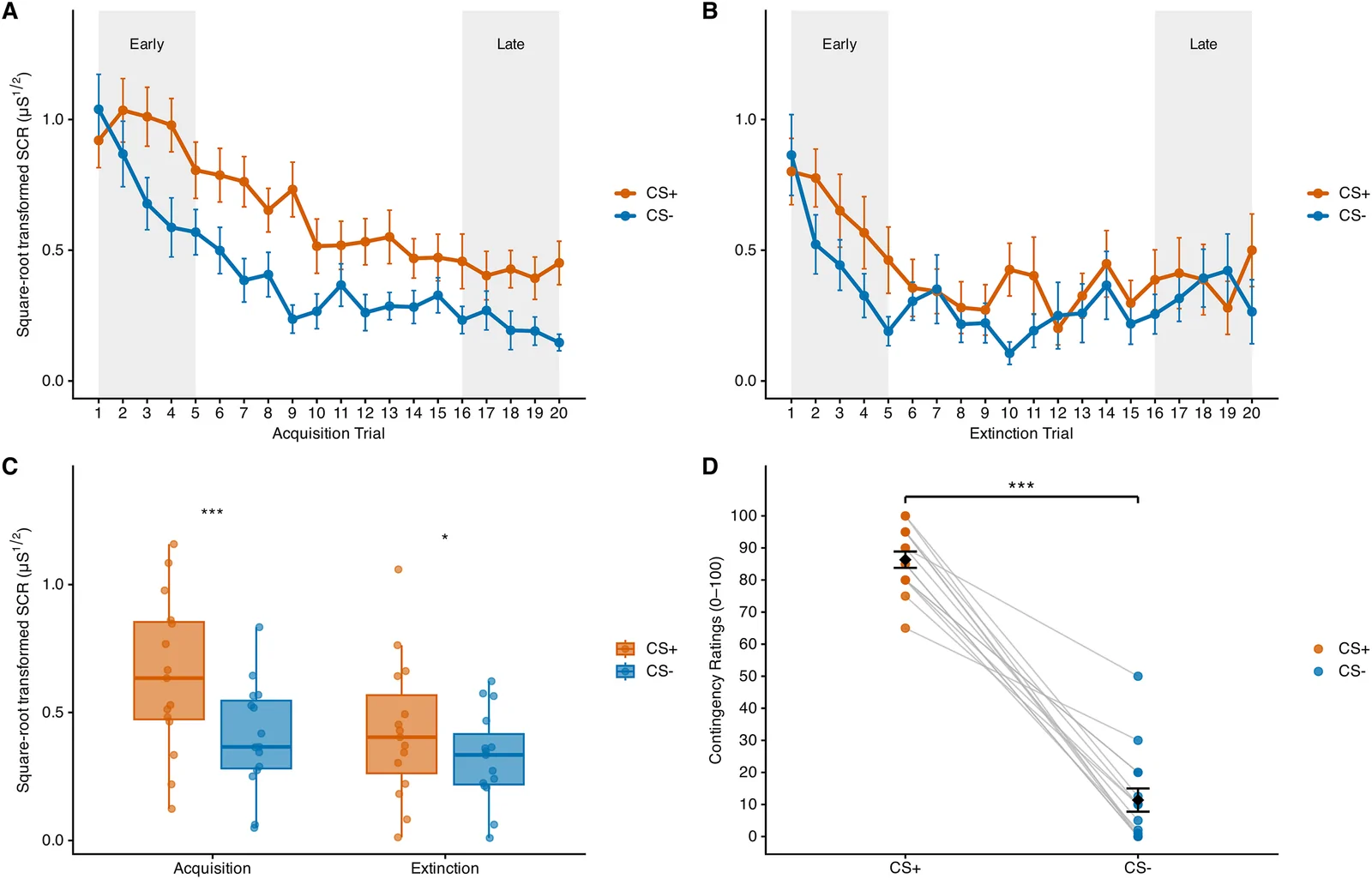
**Skin conductance responses and contingency awareness during fear conditioning (A)** The plot displays square root transformed SCR for conditioned stimuli paired with an electric shock (CS+) and never-paired (CS−) over the 20 trials during fear acquisition. Error bars indicate the standard error of the mean (SEM). **(B)** Fear extinction plotted across repeated non-reinforced CS+ and CS− presentations. The early (Trials 1-5) and late (Trials 16–20) trials are shown by gray squares. Error bars show the SEM. **(C)** Boxplots show CS+ and CS− with individual participant data for acquisition and extinction learning trials. Each box represents the interquartile range (25th–75th percentiles), the horizontal line marks the median, and whiskers extend to 1.5× the interquartile range. Two-sided paired *t*-tests were used to perform statistical comparisons. **(D)** Paired dot plot showing post-extinction contingency ratings (0–100) for CS+ and CS−. Each dot represents an individual participant, and gray lines connect paired observations from the same participant. The group mean with error bars representing the SEM is also shown. Asterisks denote statistically significant differences (***p < 0.001, *p < 0.05).

We first analysed the fear acquisition phase trial-by-trial using a linear mixed-effects model, showing greater SCR responses to CS + than CS− (t = 5.56, p < 0.001), alongside a decline in SCR over trials (t = −13.87, p < 0.001), but no stimulus × trial interaction (t = 0.08, p = 0.937) (Fig. 1A, Table S1). The aggregated measure showed greater differential SCR to CS + than CS− across all trials (mean ± SEM 0.24 ± 0.03), t (14) = 7.19, p < 0.001, Cohen's d_z_ = 1.86, 95% CI [0.17, 0.31], Fig. 1C. Baseline D2/3 receptor availability did not predict fear acquisition (Table S1). All participants exhibited contingency awareness as evidenced by higher ratings of certainty to receive an electric shock (US) to the CS+ (M = 86.33, SD = 9.90) than to the CS− (M = 11.37, SD = 14.01) (t (14) = 14.85, p < 0.001, Cohen's dz = 3.83) (Fig. 1D). Hence, there was evidence of fear acquisition but with no significant association to baseline D2/3 receptors availability.

#### 3.1.2 Fear extinction

We then focused on the extinction phase, where SCRs were significantly greater for the CS + compared to the CS− (t = 2.46, p = 0.014), with a significant general decrease in SCR across extinction trials (t = −2.66, p = 0.008), but no stimulus × trial interaction (t = −0.98, p = 0.327) (Table 1). For the aggregated differential SCR measure, we detected greater SCR to CS + than CS− across all trials ($\Delta {\text{SCR}}_{All\;Trials}$ = mean ± SEM 0.1 ± 0.04), t (14) = 2.45, p = 0.028, Cohen's d_z_ = 0.63, 95% CI [0.01, 0.19], Fig. 1C).

**Table 1:** Fixed effects from linear mixed-effects models examining trial-by-trial skin conductance responses (SCR) during fear extinction learning and its associations with baseline D2/3 receptor availability in the amygdala, dorsal striatum, and ventral striatum, as well as for SCR related to omission of the aversive unconditioned stimulus (US) during extinction.

Predictors	β	SE	t	p
**Extinction SCR**				

CS: CS+	0.1601	0.0651	2.46	0.014*
trial	−0.0102	0.0038	−2.66	0.008**
CS+ × trial	−0.0053	0.0054	−0.98	0.327

**Extinction SCR, baseline D2/3 receptor availability**				

**Amygdala**				

CS: CS+	0.4381	0.3589	1.22	0.223
trial	−0.0124	0.0212	−0.59	0.558
amygdala D2/3	−1.5626	0.8407	−1.86	0.069
CS+ × trial	0.0001	0.0300	0.00	0.998
CS+ × amygdala D2/3	−0.7066	0.8970	−0.79	0.431
trial × amygdala D2/3	0.0056	0.0529	0.11	0.915
CS+ × trial × amygdala D2/3	−0.0137	0.0749	−0.18	0.855

**Dorsal striatum**				

CS: CS+	0.6842	0.5501	1.24	0.214
trial	−0.0500	0.0325	−1.54	0.124
dorsal striatum D2/3	−0.5792	0.2826	−2.05	0.047*
CS+ × trial	−0.0276	0.0459	−0.60	0.549
CS+ × dorsal striatum D2/3	−0.2656	0.2768	−0.96	0.338
trial × dorsal striatum D2/3	0.0202	0.0163	1.23	0.218
CS+ × trial × dorsal striatum D2/3	0.0113	0.0231	0.49	0.626

**Ventral striatum**				

CS: CS+	0.1348	0.4548	0.30	0.767
trial	−0.0549	0.0268	−2.04	0.041*
ventral striatum D2/3	−0.7154	0.3489	−2.05	0.048*
CS+ × trial	0.0060	0.0380	0.16	0.874
CS+ × ventral striatum D2/3	0.0184	0.3300	0.06	0.956
trial × ventral striatum D2/3	0.0328	0.0195	1.68	0.093
CS+ × trial × ventral striatum D2/3	−0.0083	0.0275	−0.30	0.763

**US omission SCR**				

CS: noUS CS+	0.1889	0.0561	3.37	<0.001***
trial	−0.0028	0.0033	−0.84	0.404
noUS CS+ × trial	−0.0121	0.0047	−2.58	0.010*

Note. Fixed-effect estimates (β), standard errors (SE), t values, and p values are shown. SCR was square-root transformed. Reference levels: CS− and noUS CS−. Subject was included as a random intercept. Significance is indicated by *p < 0.05, **p < 0.01.

#### 3.1.3 Association between D2/3 availability and fear extinction

Contrary to our initial hypothesis, baseline D2/3 receptor availability in the amygdala, dorsal striatum, and ventral striatum were not significantly associated with trial-by-trial changes in differential conditioned responding during fear extinction, as indicated by non-significant stimulus × trial × baseline D2/3 interactions for the LME models (Table 1). For the aggregated SCR measures, baseline dopamine D2/3 receptors in neither of these regions were significantly associated with initial conditioned fear expression during early extinction, residual fear during late extinction, nor extinction-related fear reduction (all ps > 0.05; Supplementary Table S2).

#### 3.1.4 US omission-related SCR

During extinction, SCR was greater to US omission following CS + than following CS−, and this differential US omission SCR decreased across extinction trials as evidenced by the LME stimulus × trial interaction (Table 1; Supplementary Fig. S1).

**Fig. 2:**
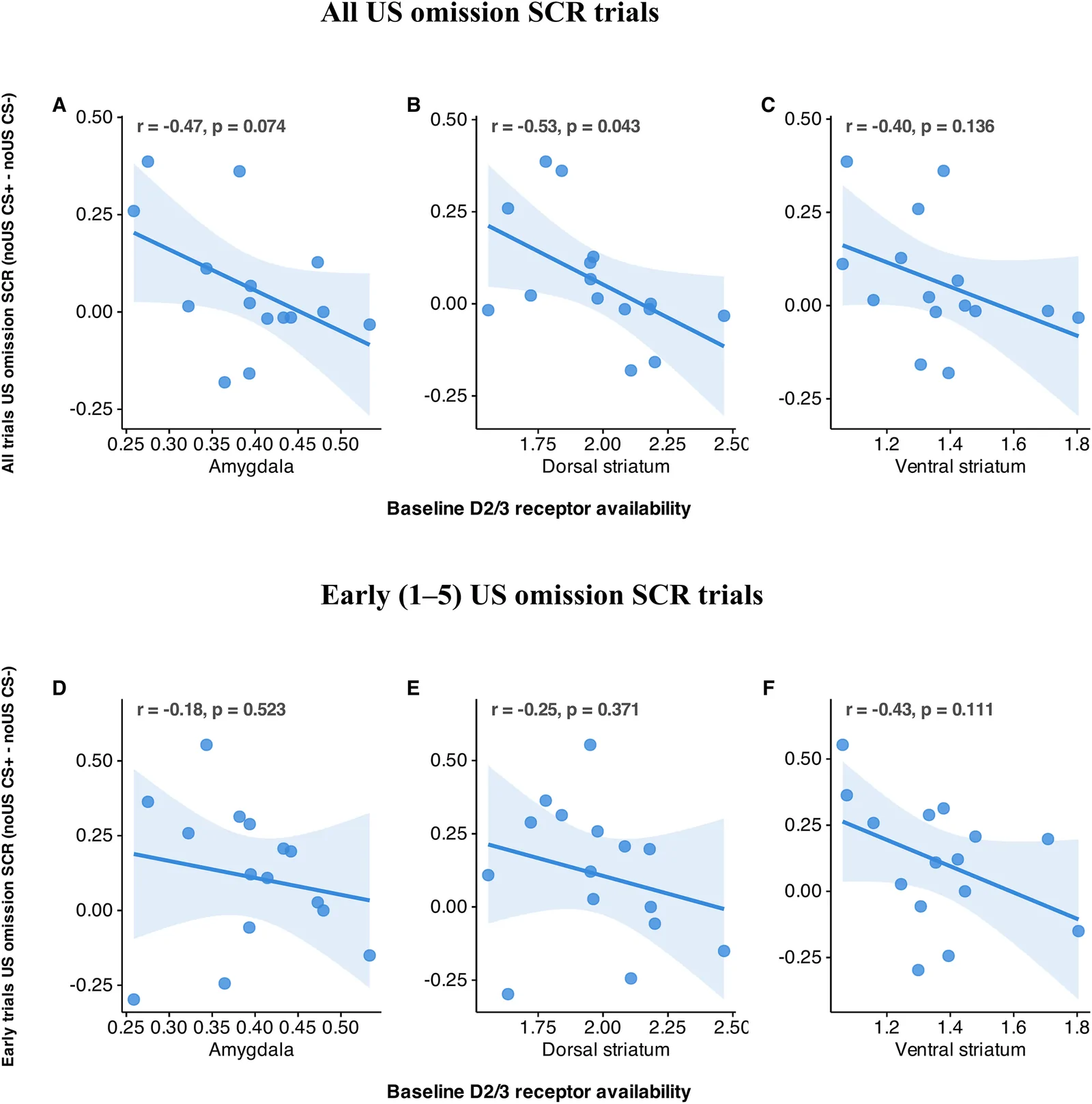
**Baseline D2/3 receptor availability and its association with omission-related SCR during extinction.** Scatter plots showing the correlations between US omission-related SCR (noUS CS + - noUS CS−) for all (top-row) and early (bottom-row) trials, a proxy measure of prediction error, and baseline D2/3 receptor availability in the **(A, D)** amygdala, **(B, E)** ventral striatum, and **(C, F)** dorsal striatum. Each dot represents a participant. The line represents the linear regression fit with 95% confidence interval (shaded). r = Pearson correlation coefficient, p = two-sided p-value.

For the aggregated differential US omission SCR measure (noUS CS+ − noUS CS−) for all trials, we found negative correlations with baseline D2/3 receptor availability in the dorsal striatum (r = −0.53, 95%, CI [−0.82, −0.02], p = 0.043) (Fig. 2). This was confirmed in a robust percentage bend correlation (r = −0.66, p = 0.007). Similar trends appeared for the amygdala and ventral striatum although they failed to reach statistical significance. Focusing on the first 5 trials, we detected no associations between the aggregated US omission SCR and baseline D2/3 receptor availability in the amygdala (r = −0.18, 95% CI [−0.63, 0.37], p = 0.52), dorsal striatum (r = −0.25, 95% CI [−0.68, 0.30], p = 0.37), or ventral striatum (r = −0.43, 95% CI [−0.77, 0.11], p = 0.111).

### 3.2 fMRI activation

#### 3.2.1 Differential responses to conditioned stimuli

**Fig. 3:**
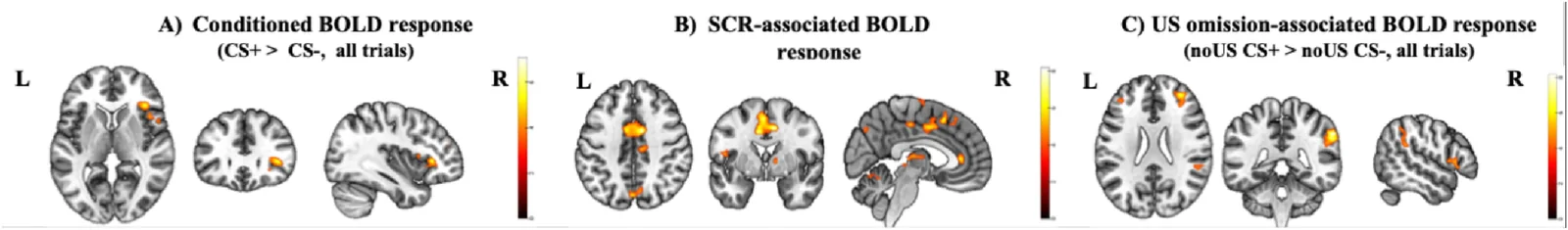
**Brain responses during fear extinction. (A)** Conditioned neural fear-response (CS+ > CS−) peak activation was detected in the supramarginal gyrus (MNI: 62, −42,36). **(B)** Differential SCR fear response (CS+ − CS−) was associated with the conditioned neural fear response (CS+ > CS−), with peak activation in the right middle cingulate cortex (MNI: 10, 0, 42). **(C)** US omission during extinction learning (noUS CS + -noUS CS−, all trials) showed significant peak activation in supramarginal gyrus (MNI: 62, −44, 40). Statistical maps were thresholded at a voxel-wise p < 0.001 (uncorrected), with cluster-level p < 0.05 family-wise error correction (FWE) and overlaid onto the MNI152 template. The color bar denotes T-values. L: Left, R: Right.

To assess neural correlates of extinction, we first examined change in differential BOLD response to CS+ > CS− across trials using parametric modulation by trial number (CS + PM > CS−PM), which detected no significant activation changes. We then tested the overall fear response across all extinction trials (CS+ > CS−), showing greater activation in the right supramarginal gyrus (SMG), middle frontal gyrus (MFG), anterior insula, and inferior frontal gyrus (IFG), pars opercularis (Fig. 3A, Table 2).

**Table 2:** Brain activations during fear extinction for the conditioned response (CS+ > CS−) and unexpected US omission (noUS CS+ > noUS CS−).

Region^a^	Hemisphere	Cluster size	MNI (mm)^b^			*Z*	p_FWE_^c^
			x	y	z		
**A) Conditioned BOLD response (CS+ > CS−, all trials)**							

Supramarginal gyrus	Right	191	62	−42	36	4.81	< 0.001
Middle Frontal gyrus	Right	110	34	40	22	4.77	0.023
Anterior Insula	Right	227	38	30	4	4.17	0.003
Inferior frontal gyrus pars opercularis	Right	139	42	16	8	3.94	0.007


**B) SCR-associated BOLD response**							

Middle cingulate cortex	Right	1095	10	0	42	4.85	< 0.001
Thalamus	Left	611	−8	−22	14	4.80	< 0.001
Supplementary motor area	Left	184	0	16	52	4.79	< 0.001
Precuneus	Left	201	−6	−66	40	4.59	< 0.001
Anterior Cingulate cortex	Right	129	8	38	14	4.45	0.006
Supramarginal gyrus	Left	113	−46	−42	24	4.38	0.012
Cerebellar (lobule VI)	Right	84	36	−62	−20	4.25	0.048
Insula	Left	238	−42	6	0	4.16	< 0.001
Middle cingulate cortex	Right	91	8	−20	44	3.85	0.034
Calcarine cortex	Right	113	20	−62	6	3.80	0.012
Cerebellar Crus II	Left	97	−6	−74	−28	3.66	0.026


**C) US omission-associated BOLD response (noUS CS+ > noUS CS−, all trials)**							

Supramarginal gyrus	Right	254	62	−44	40	4.83	< 0.001
Middle frontal gyrus	Right	435	34	44	22	4.72	< 0.001
Inferior frontal gyrus, orbital	Right	338	34	32	−4	4.45	< 0.001
Middle frontal gyrus	Left	95	−42	42	28	3.97	0.05

Note:

a Labels are assigned using the AAL3 atlas embedded in MRIcroGL.

b Coordinates are reported in Montreal Neurological Institute (MNI) 152 space (x, y, z; mm) and indicates the peak voxel within each cluster.

c Statistical threshold: voxel-wise threshold of p < 0.001 (uncorrected), followed by cluster-level FWE correction applied at p < 0.05. Conditioned stimulus paired (CS+) and not paired (CS−) with an electric shock during fear acquisition.

The neural correlates of differential SCR (CS+ − CS−) during extinction learning (CS+ > CS− contrast) showed positive associations in the right middle cingulate cortex, left thalamus, right ACC, insula, cerebellar Crus II and several other regions (Fig. 3B–Table 2, Supplementary Fig. S2). We did not detect any associations between baseline D2/3 receptor availability and change in BOLD response over extinction learning (PM approach) or with the overall fear response across all extinction trials.

#### 3.2.2 Responses to the unexpected omission of the US

For the US omissions (noUS CS+ > noUS CS−) across all trials, significant peak activations were found in the right SMG, and bilateral MFG, and the orbital part of right IFG (Fig. 3C–Table 2). We did not detect any significant activations when focusing on the first 5 trials of US omissions or for the change in BOLD response to US omission over the extinction task (PM approach), or any associations between baseline D2/3 receptor availability and US omission BOLD for all trials, the first 5 trials, or the change over the extinction task.

### 3.3 fMRI functional connectivity

#### 3.3.1 Connectivity related to conditioned responding

To examine functional connectivity during extinction, we first tested the aggregated CS+ > CS− connectivity across all trials, which did not yield any significant results. We then tested if functional connectivity changed over the course of the extinction task using the trial-wise parametric modulation approach, finding no significant changes.

**Fig. 4:**
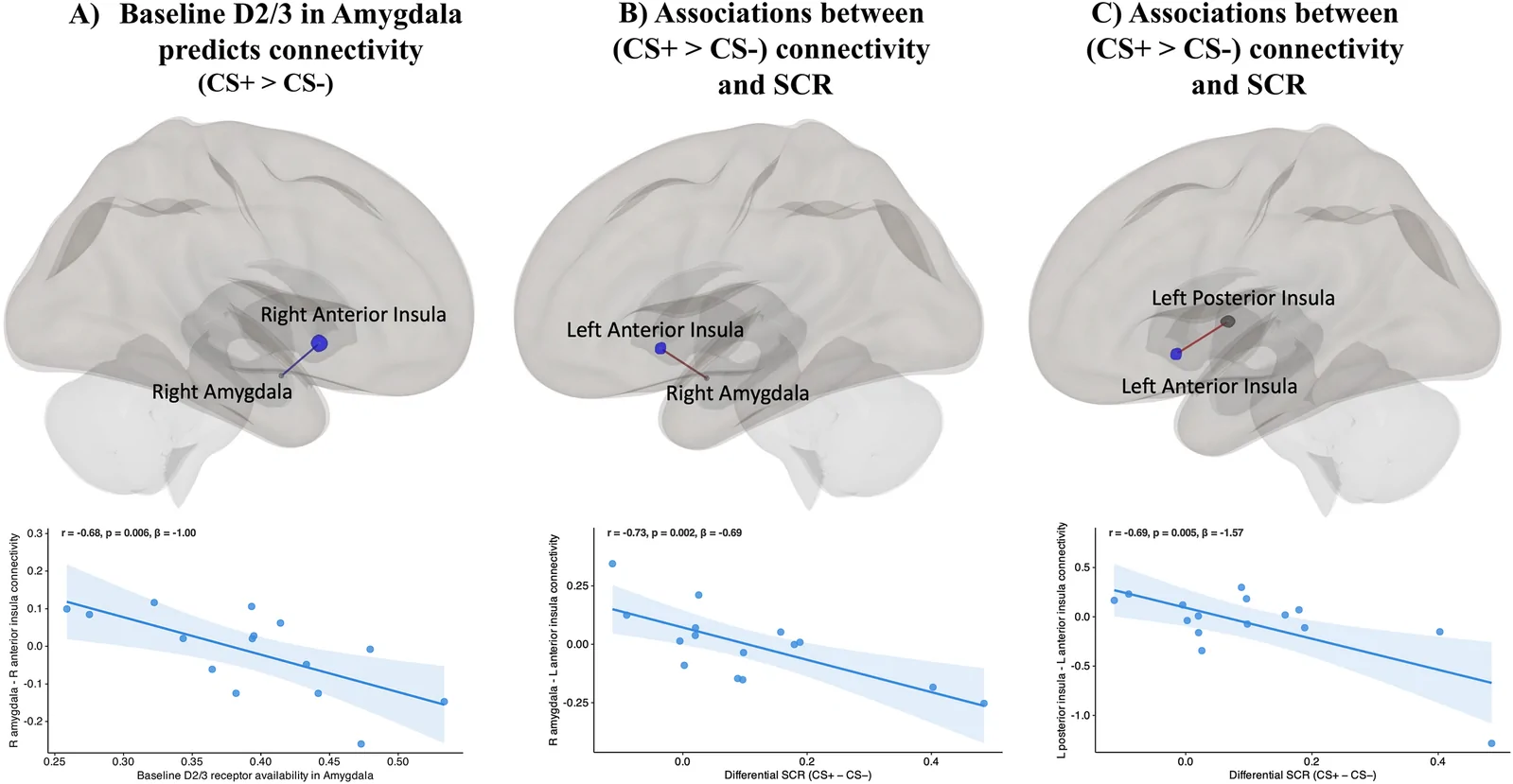
**Associations between fear-related connectivity and baseline D2/3 availability and SCR. (A)** Higher levels of baseline dopamine D2/3 receptor availability in the amygdala were associated with lower conditioned fear-related functional connectivity (CS+ > CS−) between the right amygdala and right anterior insula during fear extinction. **(B)** Differential SCR (all Trials) was related to decreased functional connectivity (CS+ > CS−) between the right amygdala and left anterior insula, and **(C)** between left posterior and anterior insula. The latter association did not survive robust correlation. Each predictor variable was correlated with the extracted ROI-ROI connectivity values and the resulting associations are displayed as scatter plots with linear regression lines; r and p-values are reported within each panel. Colored lines indicate average connectivity (red: positive, blue: negative); colored spheres represent significant connectivity-covariate (D2/3 or SCR) associations (red: positive β; blue: negative β). All results are thresholded at p < 0.001 uncorrected and FDR-corrected at the connection level (p < 0.05).

Baseline D2/3 receptor availability in the amygdala was associated with fear-related connectivity (CS+ > CS−), specifically predicting reduced connectivity between the right amygdala and right anterior insula (beta = −1.00, T = −3.31, p-FDR = 0.039) (Fig. 4A). We extracted subject-specific right amygdala and left anterior insula connectivity estimates from the ROI-to-ROI analysis for the CS+ > CS− contrast and examined their association with baseline amygdala D2/3 availability using Pearson's correlation (r = −0.68, 95% CI [−0.88, −0.25], p = 0.006). This relationship was confirmed by a robust percentage-bend correlation (r = −0.68, p = 0.005). This indicates that individual differences in fear-related connectivity are related to D2/3 receptor availability in amygdala. Baseline D2/3 availability in the dorsal and ventral striatum were not associated with connectivity changes to CS + vs CS−.

We then tested if differential SCR (CS+ – CS–) was associated with functional connectivity across all trials and found that individuals with higher differential SCR showed lower coupling between the right amygdala and the left anterior insula (beta = −0.69, T = −3.84, p-FDR = 0.014). See Fig. 4B. We then extracted and examined the association between right amygdala and left anterior insula connectivity estimates for the CS+ > CS− contrast and differential SCR (all trials) using Pearson's correlation (r = −0.73, 95% CI [−0.90, −0.35], p = 0.002). A robust percentage-bend correlation confirmed this pattern (r = −0.68, p = 0.006).

We also detected an association between SCR and the connectivity between left anterior and posterior insula (beta = −1.57, T = −3.40, p-FDR = 0.033). We then examined the corresponding correlation (r = −0.69, 95% CI [−0.89, −0.27], p = 0.005, See Fig. 4C); however, this association was not confirmed when using a robust percentage-bend correlation (r = −0.49, p = 0.065).

**Fig. 5:**
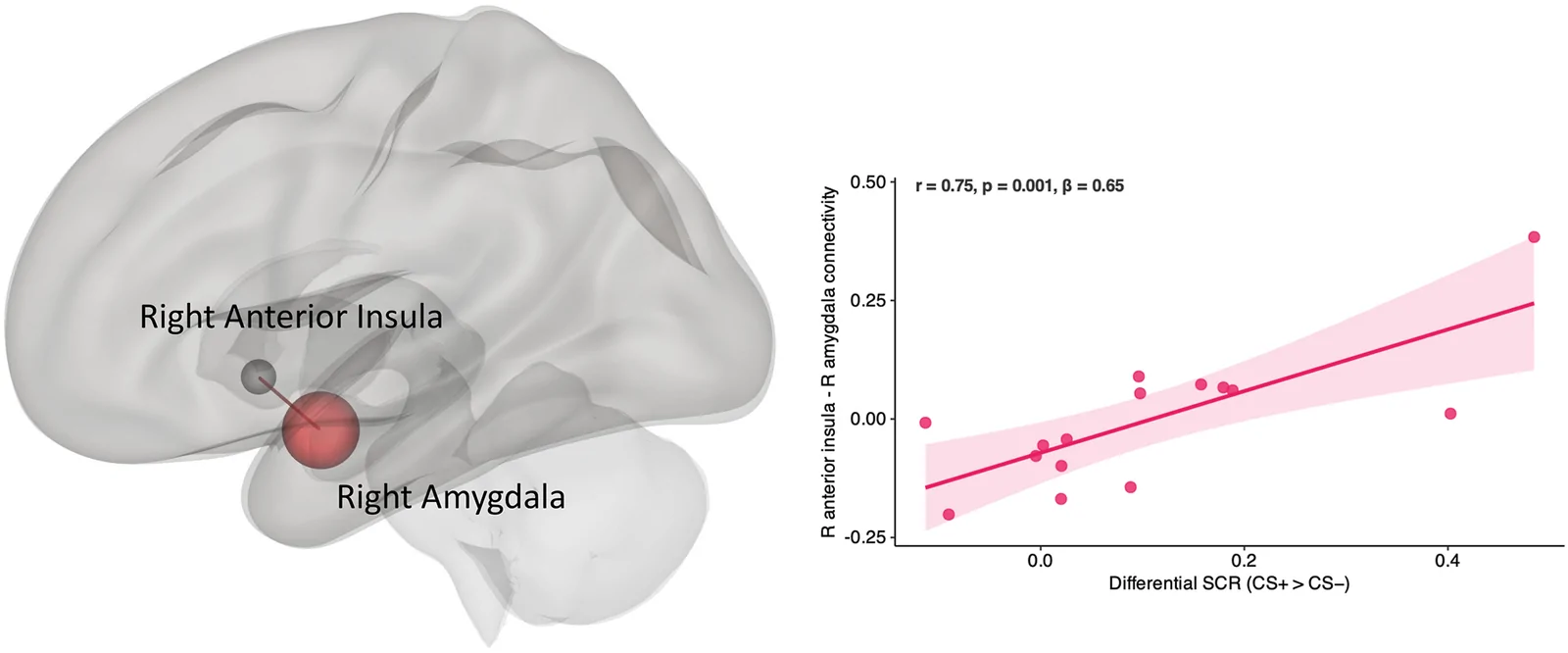
**Association between conditioned skin conductance responses and change in functional connectivity.** Higher differential SCR (CS+ − CS−) was associated with an increase in right anterior insula and right amygdala connectivity over extinction trials. The scatter plot shows the correlation; the line indicates the linear fit with its 95% confidence interval.

We also found that, during the extinction task, higher differential SCR (CS+ − CS−) was associated with a greater increase in right anterior insula and right amygdala connectivity (beta = 0.65, T = 4.12, p-FDR = 0.008) (Fig. 5), assessed using trial number as parametric modulator (CS+ > CS− × Trial number). Connectivity estimates were extracted and plotted against differential SCR (r = 0.75, 95%, CI [0.39, 0.91], p = 0.001). A robust percentage-bend correlation confirmed this pattern (r = 0.67, p = 0.006).

#### 3.3.2 Connectivity related to US omission

**Fig. 6:**
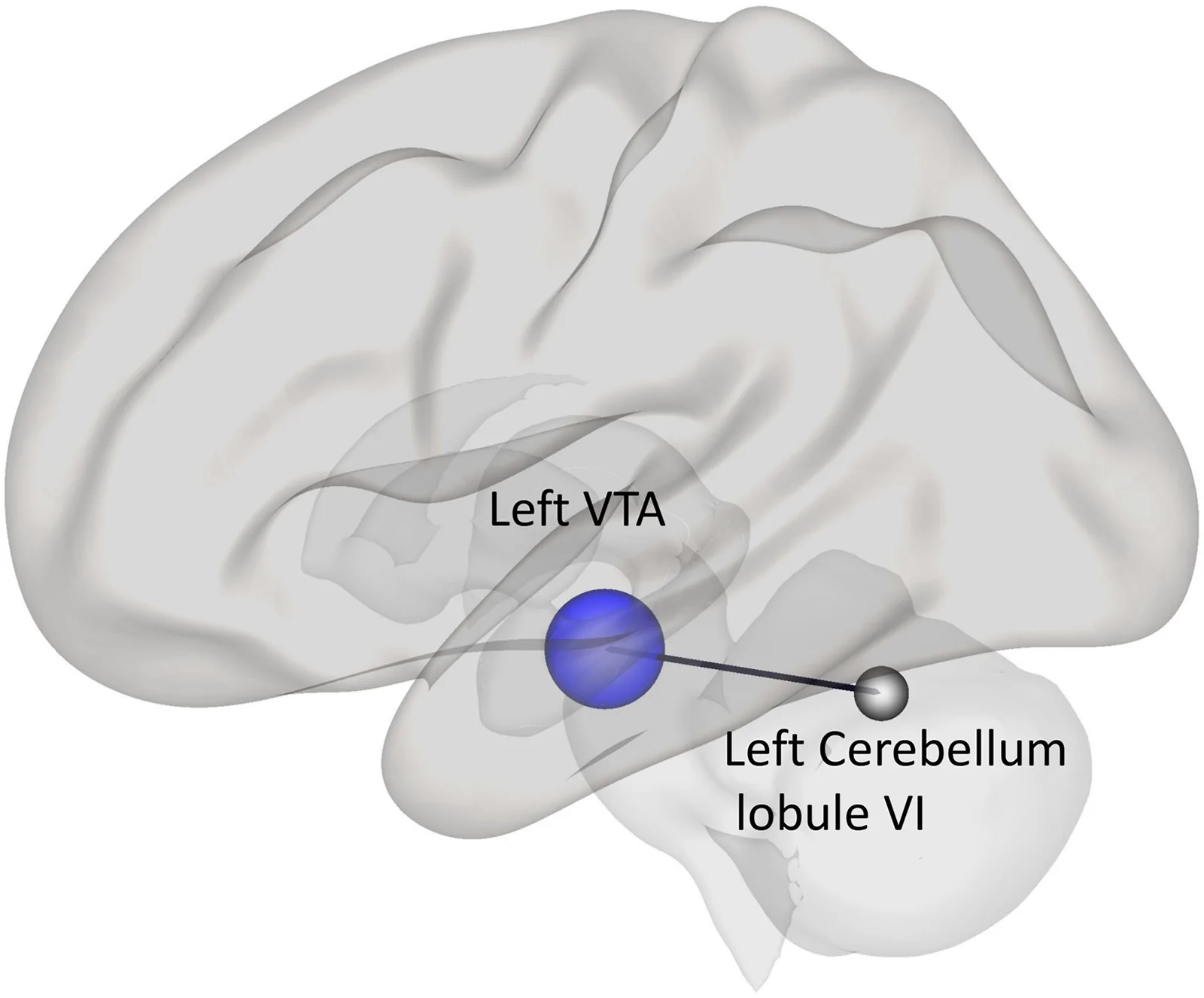
**Trial-wise modulation of cerebellum-VTA connectivity during US omission**. The omission of the US (no-US CS+ > no-US CS−) elicited significant trial-by-trial reductions in connectivity between the left cerebellar lobule VI and VTA, indicating progressively reduced coupling as extinction progressed. Note. Significance was defined as p-FDR < 0.05.

We detected no connectivity related to the US omission when examining all trials, but the trial-by-trial (parametrically modulated) omission contrast (noUS CS+ > noUS CS−) elicited a significant reduction in connectivity between the left cerebellar lobule VI and VTA (beta = −17.29, T = −3.49, p-FDR = 0.025), indicating progressively reduced coupling as extinction progressed. See Fig. 6. We detected no associations between dopamine D2/3 receptor availability and US omission-related connectivity or its change over trials.

## 4 Discussion

Here, we used skin conductance and fMRI measures from a fear extinction task in combination with PET to investigate whether interindividual differences in baseline dopamine D2/3 receptor availability relate to conditioned fear responses, extinction learning, and responses to US omission. We found that higher baseline D2/3 receptor availability in the amygdala was associated with lower fear-related functional connectivity between the right amygdala and right anterior insula during extinction. In addition, higher differential SCR (CS+ − CS−), a proxy of fear expression, was related to lower fear-related right amygdala to left anterior insula connectivity, suggesting a relationship between fear expression and salience/interoceptive network organization. Finally, lower D2/3 receptor availability was associated with larger SCR to unexpected US omissions. Together, these findings suggest that individual differences in dopamine D2/3 receptor availability are related to neural and physiological processes engaged during fear extinction, although we detected no associations with extinction-related reductions in conditioned responding per se.

We found significant associations between conditioned SCR and neural fear responses (CS+ − CS−) in the insula, ACC, mid cingulate, thalamus, supplementary motor area, precentral gyrus, precuneus, SMG, calcarine cortex, cerebellar lobule VI and cerebellar Crus II. These activations may reflect heightened anticipatory processing and threat appraisal in response to the CS + stimulus and are in line with recent meta-analytic findings (Fullana et al., 2016, 2018; Radua et al., 2025). Previous research has shown that insula activation reflects stimulus-outcome contingencies during fear conditioning, and that this effect emerges only when individuals are aware of the CS−US relationship (Tabbert et al., 2011). More recently, this has been extended by demonstrating that anterior insula activity progressively decreases during extinction training, in parallel with reduced US-expectancy and arousal (Ridderbusch et al., 2021), suggesting that the insula dynamically tracks the updating of threat predictions over time.

Notably, we found a negative association between baseline D2/3 availability in the amygdala and functional connectivity between the right amygdala and anterior insula during fear extinction. This finding is of particular interest in light of preclinical evidence indicating that amygdala D2 receptor signaling contributes to fear extinction, whereas pharmacological blockade of D2 receptors disrupts extinction learning (Shi et al., 2017). Given the established roles of these regions in salience detection (Menon and Uddin, 2010) and threat processing (J. E. LeDoux, 2000; Phelps and LeDoux, 2005), this finding suggests that individual differences in D2/3 receptor availability may be related to variation in how threat-related information is represented and communicated within salience-related circuits during extinction.

Furthermore, higher differential SCR (CS+ − CS−), was associated with reduced fear-related functional connectivity between the right amygdala and left anterior insula connectivity, as well as reduced intra-insular connectivity, although the latter did not survive robust correlation analysis. These findings might suggest that individuals with stronger fear response may exhibit diminished average connectivity both between and within core regions of the salience network, alongside elevated SCR during fear extinction, reflecting altered integration of emotional signals. The posterior-to-anterior gradient of interoceptive integration (Craig, 2009; Namkung et al., 2017), suggests that the posterior insula encodes raw bodily states and the anterior insula integrates them into emotional awareness. The association between reduced connectivity across this axis and higher fear expression may indicate impaired integration of interoceptive signals during extinction learning. This neural pattern may indicate residual fear or reflect neural activation responses similar to those observed during fear acquisition (Fullana et al., 2018; Radua et al., 2025; Vinberg et al., 2022).

The similarity is further supported by the SCR and neural activity findings, showing larger responses to the shock-associated CS+ than to the CS− during the fear extinction phase, without robust signs of attenuated fear responses. The generally higher SCR to CS+ than CS− across extinction trials may indicate either persistent fear expression, or initial extinction and subsequent rebound of fear responses. The lack of extinction-related decline in response may be attributable to the use of immediate extinction, as opposed to delayed extinction, which permits consolidation of fear memory prior to extinction learning. Since the extinction phase occurred approximately 30 min following acquisition, it is likely that the fear memory remained in a labile state while the competing safety memory had not yet consolidated, thereby promoting the continued expression of fear responses. Similarly, across extinction trials, we also observed that fear-related SCR was associated with progressively stronger connectivity between the right anterior insula and right amygdala. The stronger increase observed in coupling in individuals with higher fear-related SCR may reflect sustained engagement of the threat-appraisal system, rather than its disengagement. One interpretation is that participants continued to appraise the CS+ as salient, even when the US was repeatedly withheld, indicative of incomplete or slow extinction learning. Prior research has also shown that strong amygdala-insula coupling is often observed during fear expression, both in healthy individuals and in those with anxiety disorders (Etkin and Wager, 2007). Evidence from rodent studies suggests that distinct neuronal pathways within the anterior insula exert opposing influences on fear expression, with stimulation of the anterior insula-amygdala pathway enhancing freezing behaviour and stimulation of the anterior insula–medial thalamus attenuating freezing (Park et al., 2024). Alternatively, a recent study found that these regions can exhibit phase-dependent multivariate representations of threat and safety (Wen et al., 2024). Therefore, their recruitment during extinction may reflet dynamic changes in threat representation or learning-related updating, rather than persistence of the conditioned response.

Omission of the US during extinction was associated with increased activations in right SMG, bilateral MFG, and the orbital part of right IFG. These regions, and particularly the orbital part of the IFG, have previously been implicated in encoding the discrepancy between prior expectations and outcomes (Sherman et al., 2016). More broadly, frontal cortical recruitment during omission has been linked to expectancy updating of learned threat predictions (Dunsmoor and LaBar, 2012). In contrast to recent reports of VTA and cerebellar involvement during US omission-related processing (Nio et al., 2026), we detected no omission-related activity in either region. However, functional connectivity between cerebellar lobule VI and VTA decreased across extinction trials, which may reflect the progressive reduction in prediction-error magnitude as omission of the US becomes increasingly expected.

In line with theories linking US omission to positive prediction error signals and dopaminergic function, lower baseline dorsal striatal D2/3 receptor availability was associated with greater SCRs to US omission. However, D2/3 receptor availability was not associated with neural measures of US omission, and we detected no associations between D2/3 receptor availability and extinction-related reductions in conditioned responding.

Thus, the present findings provide only limited evidence for dopaminergic involvement in extinction learning itself. Instead, they suggest that individual differences in D2/3 receptor availability may be related to the physiological processing of expectancy violation during extinction. One possibility is that variation in D2/3 receptor availability influences the physiological processing of omitted aversive outcomes, thereby contributing to interindividual differences in the updating of threat expectations. Consistent with pharmacological evidence indicating modest and mixed evidence of dopaminergic modulation on fear extinction in humans (Doubliez et al., 2025; Gerlicher et al., 2019; Kalisch et al., 2019), our findings support a view of the dopaminergic system's role in fear extinction learning as more multifaceted than previously discussed from rodent models (Sartori et al., 2024).

Taken together, these findings suggest that individual differences in baseline D2/3 receptor availability are more strongly related to neural and physiological processing of threat and expectancy violation than to extinction learning itself. Although baseline D2/3 receptor availability was associated with fear-related amygdala-insula connectivity and omission-related SCR, associations with extinction-related changes in conditioned responding, brain activity, and functional connectivity were largely absent. This pattern suggests a more selective role for D2/3 receptors in fear extinction than proposed by several preclinical models.

### 4.1 Limitations

While the current study provides important insights into the role of dopamine in fear extinction learning, several limitations should be acknowledged. First, the sample size was relatively small, which may have precluded the detection of small to moderate effects and may have led to inflated effect sizes for findings that survived thresholds of statistical significance. Future studies should replicate the findings in larger samples. Second, participants underwent the extinction phase approximately 30 min after fear acquisition, thus not allowing for consolidation of fear memories before extinction. Immediate and delayed extinction may engage partially distinct mechanisms, limiting the generalization of our findings to delayed extinction and clinical settings, where exposure therapy targets more long-term memories. Furthermore, extinction retention was not assessed, preventing conclusions regarding the role of D2/3 receptor availability in long-term extinction memory or extinction recall. Moreover, given the small sample size, trial number was employed as a first-order (linear) parametric modulator to characterize changes in conditioning- and omission-related BOLD responses across trials. However, extinction learning may exhibit non-linear dynamics and inter-individual differences, which could be better captured using higher-order polynomial functions or computational model-derived modulators, rather than assuming a uniform trajectory for all participants. Additionally, we examined only one component of dopamine signaling, baseline D2/3 receptor availability. Although PET-derived baseline D2/3 receptor availability provides an in vivo measure of interindividual variation in the dopaminergic system, binding potential does not differentiate among receptor density, receptor affinity, or endogenous dopamine levels. Consequently, the observed associations should be considered indicative of variability in D2/3 receptor availability rather than direct measures of dopamine signalling or receptor activation. Further studies should complete the picture with measures of D1 receptors and endogenous dopamine release during fear extinction. Finally, participants self-reported as healthy rather than undergoing formal clinical assessment. Therefore, undiagnosed health conditions cannot be fully excluded.

## 5 Conclusion

In conclusion, the present study combined PET measures of baseline dopamine D2/3 receptor availability with fMRI and physiological measures during fear extinction in humans. Our findings suggest that individual variation in dopamine D2/3 receptor availability is associated with specific neural and physiological responses during extinction, particularly those related to threat processing and the unexpected omission of aversive outcomes. These findings contribute to a growing body of evidence suggesting that the role of dopamine in human fear extinction is complex and may be more evident in processing of threat and expectancy violation than in extinction learning itself. Future studies with larger samples, delayed extinction designs, extinction-retention tests, and broader measures of dopamine function (e.g., dopamine release and D1 receptors) are essential to further elucidate these mechanisms.

## CRediT authorship contribution statement

**Ashika Anne Roy:** Writing – review & editing, Writing – original draft, Methodology, Formal analysis, Data curation, Conceptualization. **Johan Vegelius:** Writing – review & editing, Supervision, Methodology. **Johannes Björkstrand:** Writing – review & editing, Methodology, Investigation, Conceptualization. **Mark Lubberink:** Writing – review & editing, Software, Methodology, Data curation. **Mats Fredrikson:** Writing – review & editing, Methodology, Investigation, Conceptualization. **Fredrik Åhs:** Writing – review & editing, Methodology, Funding acquisition, Conceptualization. **Andreas Frick:** Writing – review & editing, Supervision, Project administration, Methodology, Investigation, Funding acquisition, Data curation, Conceptualization.

## Declaration of generative AI and AI-assisted technologies in the writing process

During the preparation of this article, ChatGPT and Microsoft Copilot were utilized to enhance the language quality in specific sections of this article. The authors have reviewed and edited the content and take the full responsibility for the published article.

## Funding

This work was funded by the 10.13039/501100004359Swedish Research Council grant 2021-03106, the Swedish Brain Foundation, and Heumanska stiftelsen. The funding organisations were not involved in the study design, data collection, data analysis, interpretation of the results, preparation of the manuscript, or the decision to submit the work for publication.

## Declaration of competing interest

The authors declare that they have no known competing financial interests or personal relationships that could have appeared to influence the work reported in this paper.

## Data Availability

Due to the sensitive nature of the data collected in this study, raw data will not be shared. Processed data and code will be shared upon reasonable request.
