# Supplementary material for: Dopamine D2/3 receptors and functional connectivity during fear extinction: A PET-fMRI study
Source: Neuroimage Rep. 2026 Sep 4;6(4):100402. doi: 10.1016/j.ynirp.2026.100402 (PMC13571913; doi:10.1016/j.ynirp.2026.100402)
Supplement: Supplementary file 1 [file mmc1.docx]

**Supplementary information**

**Dopamine D2 receptors and functional connectivity during fear extinction:**

**A PET-fMRI study**

Ashika Anne Roy^1^, Johan Vegelius^1,2^, Johannes Björkstrand^3^, Mark Lubberink^4^, Fredrik Åhs^5^, Mats Fredrikson^2^, Andreas Frick^1,2^

^1^Department of Medical Sciences, Experimental Cognitive and Affective Neuroscience Lab, Uppsala University, Uppsala, Sweden

^2^Department of Psychology, Uppsala University, Uppsala, Sweden

^3^Department of Psychology, Lund University, Lund, Sweden

^4^Department of Surgical Sciences, Nuclear Medicine & PET, Uppsala University, Uppsala, Sweden

^5^Department of Psychology and Social Work, Mid Sweden University, Östersund, Sweden

**Correspondence to**:

Ashika Anne Roy

Department of Medical Sciences

Uppsala University Hospital, Entrance 10

751 85 Uppsala

SWEDEN

email: ashika.roy@uu.se

Telephone: +46 18 471 00 00

**Supplementary**

**Figure S1.** **Trial-wise skin conductance responses during unexpected US omission.** Mean baseline-corrected, square-root-transformed skin conductance responses (SCRs) are shown across US-omission trials for the CS+ and CS−. Lines indicate condition means, points represent individual trial means, and error bars denote the standard error of the mean. The grey shaded region denotes the early omission period comprising trials 1–5.

**Table S1**. Fixed effects from linear mixed-effects models examining associations between baseline D2/3 receptor availability and trial-by-trial skin conductance responses during fear acquisition.

| **Predictors** | **β** | **SE** | **t** | **p** |
| --- | --- | --- | --- | --- |
| **Acquisition SCR** | | | | |
| CS: CS+ | 0.2362 | 0.0425 | 5.56 | **<.001***** |
| trial | -0.0348 | 0.0025 | −13.87 | **<.001***** |
| CS+ × trial | 0.0003 | 0.0035 | 0.08 | 0.937 |
|  |  |  |  |  |
| **Acquisition SCR, baseline D2/3 receptor availability** | | | | |
| **Amygdala** | | | | |
| CS: CS+ | 0.1345 | 0.2343 | 0.57 | 0.566 |
| trial | −0.0649 | 0.0138 | −4.69 | **< 0.001** |
| amygdala D2/3 | −1.8204 | 0.9649 | −1.89 | 0.075 |
| CS+ × trial | 0.0249 | 0.0196 | 1.28 | 0.203 |
| CS+ × amygdala D2/3 | 0.2584 | 0.5855 | 0.44 | 0.659 |
| trial × amygdala D2/3 | 0.0764 | 0.0346 | 2.21 | **0.027*** |
| CS+ × trial × amygdala D2/3 | −0.0627 | 0.0489 | −1.28 | 0.200 |
|  |  |  |  |  |
| **Dorsal striatum** | | | | |
| CS: CS+ | −0.0528 | 0.3613 | −0.15 | 0.884 |
| trial | −0.0423 | 0.0213 | −1.98 | **0.048*** |
| dorsal striatum D2/3 | −0.3903 | 0.2990 | −1.31 | 0.208 |
| CS+ × trial | 0.0362 | 0.0302 | 1.20 | 0.231 |
| CS+ × dorsal striatum D2/3 | 0.1464 | 0.1818 | 0.81 | 0.421 |
| trial × dorsal striatum D2/3 | 0.0038 | 0.0107 | 0.35 | 0.724 |
| CS+ × trial × dorsal striatum D2/3 | −0.0182 | 0.0152 | −1.20 | 0.231 |
|  |  |  |  |  |
| **Ventral striatum** | | | | |
| CS: CS+ | 0.1907 | 0.2975 | 0.64 | 0.522 |
| trial | −0.0670 | 0.0176 | −3.81 | **<.001***** |
| ventral striatum D2/3 | −0.5958 | 0.3590 | −1.66 | 0.114 |
| CS+ × trial | 0.0198 | 0.0248 | 0.80 | 0.426 |
| CS+ × ventral striatum D2/3 | 0.0333 | 0.2158 | 0.15 | 0.877 |
| trial × ventral striatum D2/3 | 0.0236 | 0.0127 | 1.85 | 0.064 |
| CS+ × trial × ventral striatum D2/3 | −0.0143 | 0.0180 | −0.79 | 0.428 |
|  |  |  |  |  |

Note. Fixed-effect estimates (β), standard errors (SE), t values, and p values are shown. SCR was square-root transformed. CS− served as the reference condition. Subject was included as a random intercept. Significance is indicated by **p <0.01, ***<.001

**Table S2**. Associations between baseline D2 receptor availability in the amygdala, dorsal striatum, and ventral striatum and differential SCR during early extinction (trials 1-5), late extinction (trials 16-20), and extinction magnitude $\Delta{SCR}_{Early -Late}$.

| **Region** | **Early extinction (trials 1-5)**  **r (p)** | **Late extinction (trials 16-20)**  **r (p)** | **Change**  $\boldsymbol{\Delta}\boldsymbol{SCR}_{\boldsymbol{Early}\mathbf{-}\boldsymbol{Late}}$  **r (p)** |
| --- | --- | --- | --- |
| Amygdala | −0.108 (0.702) | −0.149 (0.597) | 0.019 (0.947) |
| Dorsal Striatum | −0.192 (0.493) | 0.028 (0.922) | −0.150 (0.594) |
| Ventral Striatum | 0.058 (0.837) | −0.008 (0.978) | 0.045 (0.873) |

Note. Values are Pearson r coefficients with p-values in parenthesis.

To further characterize the relationship between neural activity and autonomic responses, we extracted BOLD signal estimates from selected regions of interest (ROIs) as depicted in Figure 3B and generated the correlation plot presented in Figure S2. Significant positive correlations were observed between differential SCR and activity in several regions implicated in emotional and autonomic processing, including the right middle cingulate cortex (r = 0.92, 95% CI [0.78, 0.97], p < .001), right anterior cingulate cortex (r = 0.90, 95% CI [0.71, 0.97], p < .001), left insula (r = 0.90, 95% CI [0.71, 0.97], p < .001), and cerebellar lobule VI (r = 0.86, 95% CI [0.63, 0.95], p < .001). These findings suggest that differential SCR is closely associated with neural activity in regions involved in the integration of affective and physiological responses.

**Figure S2.**  **Associations between differential skin conductance responses (CS+ > CS−) and regional BOLD response.** Scatterplots show the relationships between differential SCR (CS+ > CS−) and activity in four regions: (A) Right middle cingulate cortex centered at MNI coordinates (10, 0, 42), (B) right anterior cingulate cortex centered at (8, 38, 14), (c) left insula centered at (−42, 6, 0), and (D) cerebellar lobule VI centered at (36, −62, −20). Differential SCR was entered as the predictor and regional activity as the outcome. Shaded areas indicate 95% confidence intervals. Pearson’s correlation coefficient (r), corresponding p value, and the re
